# Switching at the ribosome: riboswitches need rProteins as modulators to regulate translation

**DOI:** 10.1038/s41467-021-25024-5

**Published:** 2021-08-05

**Authors:** Vanessa de Jesus, Nusrat S. Qureshi, Sven Warhaut, Jasleen K. Bains, Marina S. Dietz, Mike Heilemann, Harald Schwalbe, Boris Fürtig

**Affiliations:** 1 Institute of Organic Chemistry and Chemical Biology - Center for Biomolecular Magnetic Resonance (BMRZ), Johann Wolfgang Goethe-University Frankfurt, Max-von-Laue Straße 7, Frankfurt am Main, Germany; 2 Institute of Physical and Theoretical Chemistry, Johann Wolfgang Goethe-University Frankfurt, Max-von-Laue Straße 7, Frankfurt am Main, Germany

**Keywords:** RNA, Solution-state NMR

## Abstract

Translational riboswitches are cis-acting RNA regulators that modulate the expression of genes during translation initiation. Their mechanism is considered as an RNA-only gene-regulatory system inducing a ligand-dependent shift of the population of functional ON- and OFF-states. The interaction of riboswitches with the translation machinery remained unexplored. For the adenine-sensing riboswitch from *Vibrio vulnificus* we show that ligand binding alone is not sufficient for switching to a translational ON-state but the interaction of the riboswitch with the 30S ribosome is indispensable. Only the synergy of binding of adenine and of 30S ribosome, in particular protein rS1, induces complete opening of the translation initiation region. Our investigation thus unravels the intricate dynamic network involving RNA regulator, ligand inducer and ribosome protein modulator during translation initiation.

## Abstract (web summary)

Translational regulation by riboswitches is an important mechanism for the modulation of gene expression in bacteria. Here the authors show that the ligand-induced allosteric switch in the adenine-sensing riboswitch from *V. vulnificus* is insufficient and leads only to a partial opening of the ribosome binding site and requires interaction with 30S-bound ribosomal protein S1, which acts as an RNA chaperone.

## Introduction

During translation initiation, messenger RNA (mRNA) and the 30S ribosome form a complex that is stabilized by interactions between mRNA, ribosomal RNA (rRNA) and ribosomal proteins (rproteins). The mRNA contains a ribosome binding site (RBS) of approx. 30 nucleotides (nt) around the start codon that includes the evolutionary conserved Shine-Dalgarno (SD) sequence 5’-GGAGGA-3’^1,2^. Translation initiation requires the coupling of the opening of mRNA secondary structure in the translation initiation region and binding of the 30S ribosome^3^. The opening of mRNA secondary structure is transient and often too short for recruiting the 30S ribosome^4^. To circumvent this short opening, the 30S is already bound to an mRNA standby site^4–7^ and shifts into place to form a specific complex with the SD-mRNA and the anti-SD-rRNA interaction only if the SD-mRNA site becomes accessible.

**Fig. 1:**
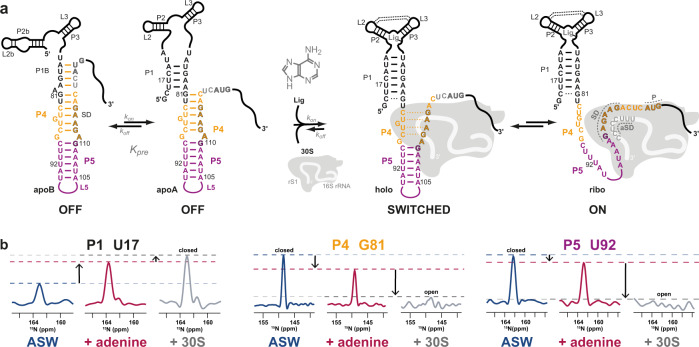
Adenine-sensing riboswitch (ASW) regulating translation initiation in the context of adenine and 30S ribosome binding. **a** The two functional OFF-conformations apoB and apoA are in a temperature-dependent equilibrium linked through *K*_pre._ Adenine binding to the aptamer region leads to a compact structure with tertiary interactions such as the kissing loop interaction between L2 and L3. 30S ribosome binding to the standby site 3′-terminal to the AUG start codon of the mRNA results in to melting of the Shine-Dalgarno (SD) sequence in helix P4 of the riboswitch (switched). Further, a stable SD-aSD interaction forms and the mRNA is incorporated into the mRNA tunnel when both P4 and P5 are opened. **b** 1D projections of the ^15^N dimension of 2D [^1^H-^15^N]-BEST-TROSY experiments at 25 °C shown for peaks in helices P1, P4 and P5 for ASW^14–140^ without (apo, blue), with 1.4 eq. adenine (holo, magenta) and in complex with the ribosome (30S + holo, gray).

Sensing of the accessibility of the SD sequence thus is one mechanism to regulate translation initiation^8^. In fact, translational riboswitches, cis-acting regulation elements that bind to a specific inducer ligand found in bacteria, modulate this accessibility^9,10^. Up to now, it is assumed that binding of an inducer ligand is sufficient for translational riboswitches to interfere with the dynamic shift between non-specific standby binding and specific binding of the 30S^9,11^. The adenine-sensing *add* riboswitch (ASW) from the human pathogenic bacterium *Vibrio vulnificus* regulates translation in an inducer ligand-dependent (characterized by the dissociation constant *K*_D_) and temperature-compensated (characterized by the equilibrium constant *K*_pre_) manner and functions over a broad temperature range^12,13^. In the absence of adenine, ASW populates two secondary structures (apoB and apoA) (Fig. 1, Supplementary Fig. 1). Both, apoB and apoA are functional OFF-states as they sequester the SD in stable base pair interactions that preclude translation initiation. The *add* riboswitch can bind adenine post-transcriptionally and operates under thermodynamic control, as shown by in vitro and in vivo studies^14^. Gene regulation of the *add* riboswitch is independent from the coupling of transcription and translation. It is clear that the kinetics of ligand binding and structural switching between apoA and apoB has to occur on a timescale faster than translation initiation^12,15^. It was shown that the rate limiting step in regulation is dependent on the RNA refolding rate, which is independent from adenine concentration^12^. Little is known about the interaction of translational riboswitches and the ribosome, since X-ray crystallography or cryo-EM studies are difficult as the regulatory relevant interactions are transient. However, NMR studies capable of detecting such transient interactions are challenging because of the size of the investigated system of more than 800 kDa, which contains the riboswitch mRNA and the 30S ribosome. Here, we succeed in applying solution NMR spectroscopy of various reconstituted adenine-sensing riboswitch-30S ribosome complexes to investigate both the influence of the inducer ligand adenine and the ribosomal modulator protein rS1 and unravel a synergistic regulatory switch to a functional ON-state.

## Results and discussion

We investigated the riboswitch (ASW^14–140^) containing 18 nucleotides within the coding region of the mRNA as a standby site for ribosome binding (Fig. 1a). The inducer ligand adenine binds to ASW with a *K*_D_ of 0.6 μM at 25 °C^12^. The helix P4 of ASW^14–140^ was only partially opened up to 20% at 25 °C, and complete opening was never reached even at saturating concentrations of the inducer ligand (Fig. 1b, Supplementary Fig. 1).

The footprint of bound 30S ribosome comprises 30 nucleotides that start 15 nt upstream of the start codon (position −15)^16^. The adenine-induced opening of RNA structure in ASW^14–140^, however, only affected nucleotides up to position −9, nucleotide A111 (Supplementary Fig. 1). This ligand-induced partial opening of P4 in ASW^14–140^ was not sufficient to initiate translation. In other words, the interaction of adenine alone with ASW^14–140^ was insufficient to enable the complete accommodation of the mRNA into the ribosome, as the neighboring helix P5 ultimately impeded binding. Therefore, we here determined the additional factors that interact with the riboswitch to enable full accommodation in the ribosomal tunnel.

### rS1 modulates riboswitch activity

**Fig. 2:**
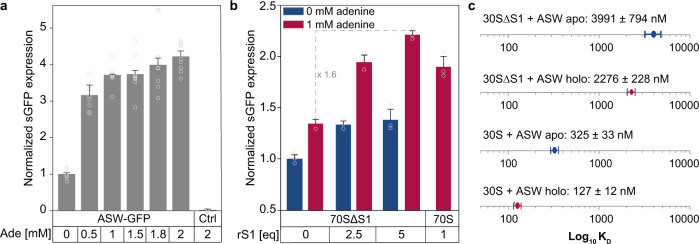
Biochemical probing of ASW function in vivo and in vitro. **a** Adenine-dependent riboswitch function in vivo. Translation efficiency was monitored by quantifying sGFP expression levels under the control of the riboswitch. For error estimation, three biological independent samples were measured in triplicates (mean ± s.d.). **b** Riboswitch function in a fully reconstituted coupled transcription-translation assay in dependence of adenine and rS1. For error estimation, one biological sample was measured in triplicates (mean ± s.d.). **c** Binding affinities of mRNA-ribosome-complexes determined by microscale thermophoresis in the absence and presence of rS1 and adenine. Source data are provided as a Source data file.

When inserted prior to the shifted green fluorescent protein (sGFP) gene, the riboswitch retained its regulatory function in vivo. Expression of sGFP under the control of the ASW led to an adenine-dependent increase of translated sGFP levels (Fig. 2a). Adenine concentrations in the range between 0.5 mM and 2 mM increased sGFP expression by ~3- to ~4-fold, respectively.

Since, the inducer ligand adenine alone could not account for a structural transition to the functional ON-state, the results of the in vivo experiments suggested that there must be additional factors of the bacterial translation machinery that positively interfered with the riboswitch. Several ribosomal proteins exhibit ATP-independent RNA chaperone activity that accelerates opening of RNA secondary structure^17,18^. Chaperone activity has been reported for rproteins rS12^17,19^, and in particular rS1^20^. rS1 is essential for translation initiation in Gram-negative bacteria^21^. Bound to the 30S ribosome^22–25^, it recruits mRNAs for translation initiation by binding to AU-rich sequences. We thus investigated the functional role of rS1 in assisting riboswitch-mediated gene regulation. rS1 concentrations cannot be easily manipulated in vivo^26^, as knock-out of rS1 is lethal^27^. Thus, we investigated its role in a reconstituted coupled transcription-translation assay (Fig. 2b) to determine rS1’s function in the riboswitch-mediated regulation of gene expression. In line with the in vivo experiments, addition of adenine resulted in an increase of sGFP expression in the presence of rS1. However, the regulation efficiency diminished to basal levels in the absence of rS1, decreasing by a factor of 1.6 upon removal of rS1. The switching behavior could be restored upon addition of rS1.

Native composite gel electrophoreses revealed complex formation of ASW^14–140^ with 30 S ribosomes (Supplementary Fig. 2). As the cognate ribosome from *V. vulnificus* is not accessible for practical and biosafety reasons, the interaction is studied here with the homologous *E. coli* ribosome. The 3′-end of the 16S rRNA in *E. coli* which harbors the aSD sequence is nearly identical (identity = 98%) to that of *V. vulnificus* (Supplementary Fig. 11) supporting the validity of using *E. coli* ribosomes to study the molecular basis of translation initiation for the *add* riboswitch from *V. vulnificus*. Binding of ASW^14–140^ to the 30S ribosome was dependent on the presence of rS1 as shown by microscale thermophoresis (MST) (Fig. 2c, Supplementary Fig. 3). The binding constants of apo and holo ASW^14–140^ to 30S∆S1 ribosomes were *K*_D_ = 4000 ± 800 nM and 2200 ± 200 nM, respectively. By stark contrast, binding of ASW^14–140^ to 30S ribosomes was substantially increased (*K*_D_ = 320 nM (apo) and 130 nM (holo)). The affinity increase was nearly four-fold larger in the holo-state hinting at synergistic structural effects of the rS1 protein and the inducer ligand adenine (Supplementary Fig. 4). Notably, the affinity of the riboswitch was further increased to *K*_D_^30S∆S1+vvrS1^ = 60 nM (holo) when the rS1 protein from *E. coli* was exchanged to rS1 from *V. vulnificus* (Supplementary Fig. 3b). To show that rS1 acts within the 30S complex, the *K*_D_ of *V. vulnificus* rS1 towards 30S was determined to be 170 nM and further NMR experiments reported on a tight binding to the 30S ribosome (Supplementary Fig. 5). The observation that adenine binds more weakly to the ASW than the ribosome hints at a structural and functional synergistic effect of the inducer ligand and the ribosome.

### 30S ribosome-bound rS1 opens expression platform in holo state

**Fig. 3:**
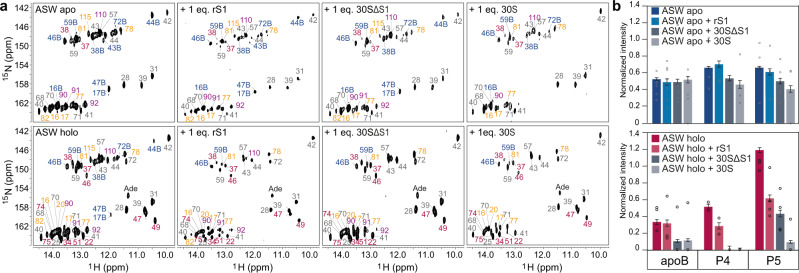
NMR spectroscopic investigations of mRNA-ribosome complexes. **a** [^1^H-^15^N]-BEST-TROSY experiments of ASW, ASW-rS1, ASW-30SΔS1 and ASW-30S in absence and presence of 1.4 eq. adenine. Peaks are color coded according to Fig. 1. **b** Normalized intensities of peaks according to the secondary structure elements. Peaks were normalized to helix P3 (see Supplementary Fig. 6, Source data are provided as a Source data file). Error bars represent the standard deviation of the average weighted by the signal-to-noise ratio of the individual peaks.

To delineate the structural basis for the increased affinity, we characterized conformational changes induced by stoichiometric amounts of rS1 and the 30S ribosome using solution NMR spectroscopy to map the base pairing interactions within ASW^14–140^ in eight different experimental setups (Fig. 3a, Supplementary Fig. 6). In the absence of inducer ligand adenine (apo state), the conformations apoB and apoA were found to be in equilibrium. In both conformations, helices P4 and P5 were closed. This structural behavior was generally maintained irrespective of the presence of rS1 alone or in the presence of the ribosomal particle without rS1 (30S∆S1). In a titration with rS1, minor effects towards a destabilization of base pairs in the helices P4 and P5 in the translation initiation region were detected at saturating levels of rS1 (Supplementary Fig. 7). Although, in absence of inducer ligand the expression platform was unresponsive against rS1 alone and 30S∆S1, it was slightly responsive against the full ribosomal particle, however the closed conformational state was still the dominant conformation (Fig. 3b). In the presence of adenine (holo state), the equilibrium was shifted away from apoB towards the holo conformation with partial opening of helix P4 and closed helix P5. By stark contrast to the apo state, the holo state of the riboswitch was much more susceptible to conformational changes upon interaction with rS1 and 30S. At 1:1 stoichiometry, addition of rS1 led to an opening of P4 and P5 by 50%. However, addition of 30S containing rS1 led to complete destabilization of the apoB conformation, complete opening of helices P4 and P5 and release of nucleotides 82–115 to adopt single strand conformation.

**Fig. 4:**
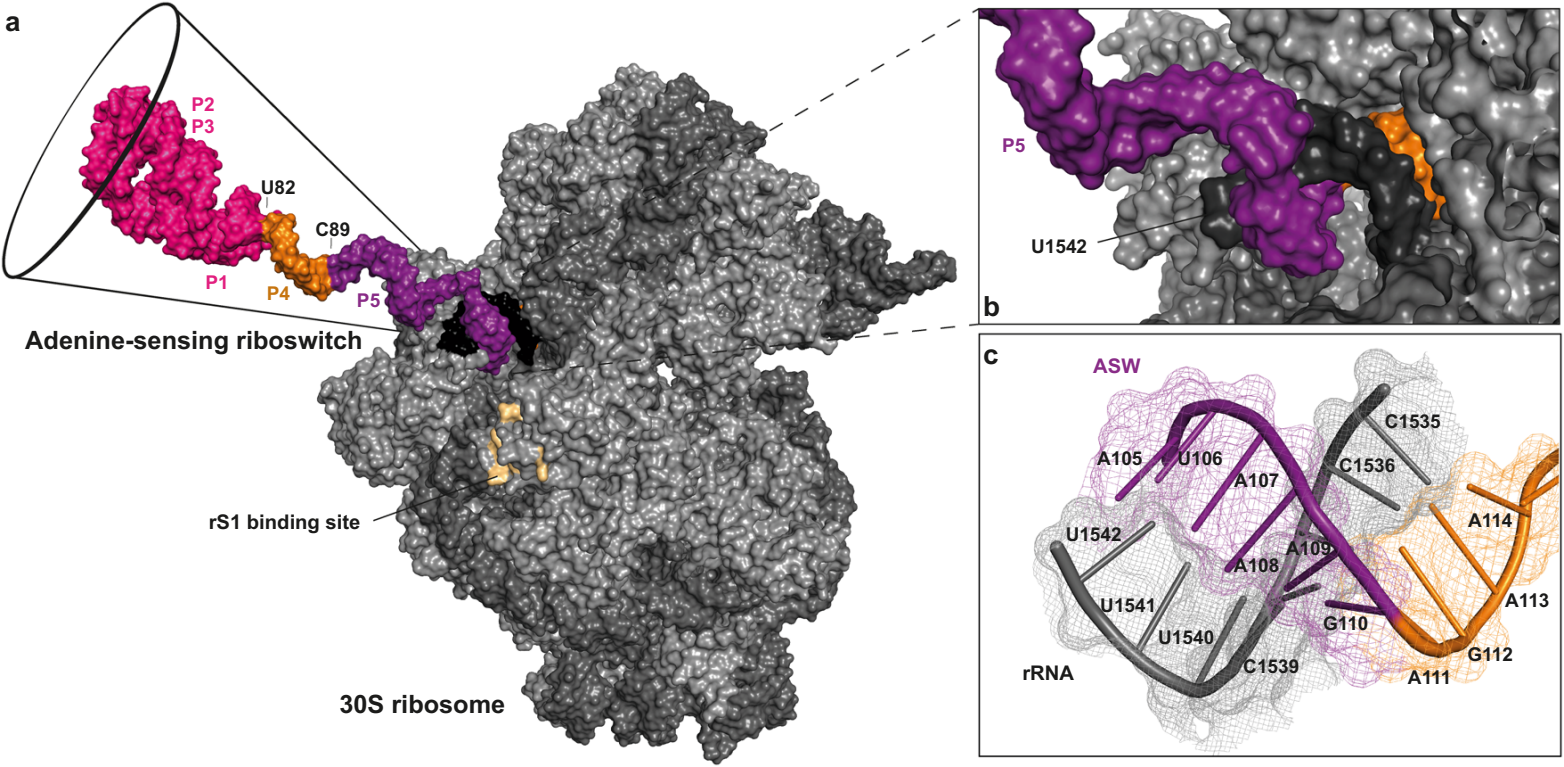
Structural model of the adenine-sensing riboswitch (ASW) in complex with 30S ribosome from *E. coli*. **a** Overall depiction of the initiating riboswitch with the 3′-end of the 16S rRNA which is shown in dark gray. The structural modules of the ASW are color-coded according to Fig. 1: aptamer (P1, P2, P3) in magenta, 5′-strand of helix P4 in orange, nucleotides of opened stem P5 in purple. The residues of rS2 forming the binding site for rS1 at the 30S ribosome are highlighted in beige^23,30^. **b**, **c** Base pairing interaction of the Shine-Dalgarno sequence of the ASW with the anti-Shine-Dalgarno sequence of the 16S rRNA.

We built a molecular model of the initiating ribosome in complex with the 5′-end of the ASW mRNA, restrained by the base pairing structure derived from the NMR experiments (Fig. 4). The effect of rS1 on opening of the translation initiation site (P4, P5 and the single stranded 3′ nucleotides) was accompanied by a stabilization of base-pairs stemming from P1, P2 and the connecting loops and junctions of ASW^14–140^, therefore the ligand bound aptamer was modeled in its compact tertiary structure. Base pairs from position 82 to 115 (P4 and P5) were molten by the 30 S complex and thus the nucleotides were kept in single stranded conformation. Consequently, the RBS including the SD sequence and neighboring secondary structure elements impeding productive mRNA binding becomes accessible for interaction with the 16S rRNA (Fig. 4c). The model showed that only if helix P5 was single stranded steric clash between r-proteins and mRNA could be avoided at the entrance of the tunnel. At this point, melting of P5 is a unique feature that only occurs through ribosome-bound rS1.

Here, rS1 exerts its RNA chaperoning function^20^ directly at the ribosome, fulfilling its role in mRNA delivery by keeping the ribosome binding site on the mRNA in a single-stranded conformation. As it is shown that rS1 promotes partial mRNA unfolding also in other riboswitches^28^, facilitating single stranded conformations within the effector domains of translational riboswitches might be a general function of rS1. However, for the ASW, the observed unfolding of this part of the riboswitch is prerequisite for productive incorporation of the mRNA into the 30S ribosome.

In summary, we found that for translational riboswitches binding of the inducer ligand stabilizes the aptamer domain and destabilizes the sequestering effect of the cis-acting complementary strand precluding SD opening. However, the presence of ligand and riboswitch RNA alone was insufficient to completely release the ribosome binding site. To achieve complete opening, the interaction with the translation machinery and in particular with the rS1 protein was essential. A translational ON-state in the complex between the riboswitch and the 30S ribosome became effectively populated only when both, the inducer ligand and the modulator rS1 were present. Only then, the SD sequence is completely released and could base pair with the aSD sequence of the 16S rRNA leading to correct positioning of the start codon. Thus, our model delineates all required components for translation initiation by riboswitches. We note that our data for the adenine-sensing riboswitch are consistent with the standby model of translation initiation^4,5,29^. Further, it challenges the “RNA-only” model of riboswitch function, as translational regulation only occurs upon concerted yet dynamic interaction of mRNA, 30S ribosomes and associated modulator proteins.

## Methods

### DNA template

The DNA template of 127 nt adenine-sensing riboswitch (ASW) from *Vibrio vulnificus* is flanked by two restriction sites and two ribozymes at both ends in order to maintain 3′- and 5′-end homogeneity:

GAATTC (EcoRI) – TAATACGACTCACTATAG (T7 Pol) – GGTATGAAGCCTGATGAGTCCGTGAGGACG AAAGACCGTCTTCGGACGGTCTC (Hammerhead ribozyme) – GCTTCATATAATCCTAATGATATGGTTT GGGAGTTTCTACCAAGAGCCTTAAACTCTTGATTATGAAGTCTGTCGCTTTATCCGAAATTTTATAAAGAGAAGACTCATGAATTACTTTGACCTGCCG (127 nucleotide ASW) – GGGTCGGCATGGCATCTCCACCTC CTCGCGGTCCGACCTGGGCTACTTCGGTAGGCTAAGGGAGAAG (HDV ribozyme) – CCCGGG (SmaI).

### Riboswitch preparation

^13^C–^15^N-labeled 127-nucleotide *add* ASW was synthesized by in vitro transcription with T7 RNA polymerase from linearized plasmid DNA^31^. The ^13^C–^15^N-labeled (rATP, rCTP, rGTP, rUTP) nucleotides for transcription were purchased from Silantes (Munich, Germany). The construct was purified by preparative polyacrylamide gel electrophoresis according to standard protocols^32^. The construct was folded in water for 5 min at 95 °C and immediately diluted 10-fold with ice-cold water. The RNA was buffer-exchanged into NMR buffer (25 mM potassium phosphate, 150 mM KCl, 5 mM MgCl_2_, pH 7.2). For protein-RNA studies, 5 mM DTT was added.

### Purification of rS1, rS1ΔD6 and rS1ΔD56

The rS1 sequence was taken from *Vibrio vulnificus* (strain CMCP6). rS1, rS1ΔD6 and rS1ΔD56 were expressed in BL21(DE3) cells carrying a N-terminal polyhistidine- and thioredoxin-tag. Cell cultures were grown in LB-medium at 37 °C. At OD_600_ 0.6–0.8, protein expression was induced with 0.5 mM isopropyl β-D-1-thiogalactopyranoside (IPTG) and incubated over night at 20 °C. The culture was harvested and resuspended in 50 mM Tris-HCl (pH 8), 300 mM NaCl, 10 mM imidazole, 10 mM β-mercapto-ethanol. The protein was purified from the soluble fraction with a 5 mL HisTrap column (GE Healthcare) using 50 mM Tris-HCl (pH 8), 300 mM NaCl, 500 mM imidazole, 10 mM β-mercapto-ethanol for elution. Tags were cleaved overnight and at 4 °C using TEV protease and removed via a second HisTrap column. The protein was further purified by size exclusion chromatography on a HiLoad 26/60 S75 column (GE Healthcare) using NMR buffer (25 mM potassium phosphate, 150 mM KCl, 5 mM DTT, pH 7.2).

### Purification of 30S ribosomes and rS1-depleted 30S ribosomes

30S ribosomes were purified based on His-tagged proteins L7/L12 following the single step protocol from Ederth et al.^33^ Briefly, *E. coli* strain JE28 was grown in LB medium supplemented with 50 µg/mL kanamycin at 37 °C. At OD_600_ 0.6, the culture was harvested by centrifugation at 4000 g for 5 min. The cell pellet was resuspended in lysis buffer (20 mM Tris–HCl pH 7.6, 10 mM MgCl_2_, 150 mM KCl, 30 mM NH_4_Cl) supplemented with 0.5 mg/mL lysozyme, 50 µL DNase 1 (2000 U/mL) and cOmplete^™^ Protease Inhibitor Cocktail tablet (Roche) and further lysed using a Microfluidizer M-110P (Microfluidics, USA) in a final volume of 50 mL. The lysate was clarified by centrifuging twice at 38420 g at 4 °C, 20 min each. For affinity purification, a HisTrap column (Ni^2+^ sepharose pre-packed, 5 mL, GE Healthcare) was connected to an ÄKTA prime chromatography system (GE Healthcare) pre-equilibrated in lysis buffer. After loading the lysate, the column was washed with 10 column volumes of 7 mM imidazole in lysis buffer. The purification of 30SΔS1 ribosomes was performed as described before. To elute the ribosomal S1 protein, the column was washed with 1.5 M NH_4_Cl in lysis buffer. To elute 30S ribosomes, a decreasing magnesium concentration gradient was applied from 10 mM to 1 mM magnesium. Fractions were concentrated to 1 mL using centrifugal filter units (Vivaspin 20, MWCO 30000) and loaded onto a sucrose gradient of 20% to 50% sucrose in sucrose gradient buffer (20 mM Tris-HCl, pH 7.6, 300 mM NH_4_Cl, 5 mM Mg(OAc)_2_, 0.5 mM EDTA and 7 mM β-mercapto-ethanol). Gradients were centrifuged for 16 h at 100000 g and 4 °C, fractionated with a Piston Gradient Fractionator (BioComp Instruments, Canada) and analyzed by SDS gel electrophoresis (Supplementary Fig. 8). 30S ribosomes were stored in sucrose buffer at 4 °C. Concentration of 30S ribosomes were determined at A_260_ where 1 A_260_ correspond to 72 pmol/mL^34^. To avoid disturbing effects of sucrose, sample concentration was determined in 1:100 dilutions after buffer exchange.

### 30S ribosome stability and integrity of NMR samples

High resolution NMR experiments require that the sample of the macromolecular complex has to be pure from contaminating proteins and nucleic acids, it must be monodispersed and due to the experimental restraints of the technique highly concentrated. Purity of the final 30S ribosomes utilized in all subsequent experiments was judged by gel-based analysis in comparison to similarly purified 50S and 70S particles (Supplementary Fig. 8).

Ribosomes were further analyzed by Western Blot using a 6x-His Tag Antibody (Thermo Fischer) that allows selective detection of the modified ribosomal protein L12. The Western Blot with this specific antibody clearly shows that 30S particles are free of impurities of 50S protein components.

Integrity of ribosomes were further analyzed by native composite gel electrophoresis and quantitative mass spectrometry (Supplementary Fig. 9). After acquiring NMR experiments, the NMR samples were analyzed for RNA integrity and 30S ribosome stability. RNA integrity was checked by denaturing polyacrylamide gel electrophoresis (Supplementary Fig. 8). Theoretically, RNA could possibly be degraded by co-purification of RNases during ribosome preparation. Ribosome stability was further analyzed by loading the measured samples on another sucrose gradient (Supplementary Fig. 10).

### Electrophoretic mobility shift assay (EMSA)

The EMSAs to investigate RNA-S1 binding were performed at constant RNA concentration (4 µM) and increasing amounts of protein (0–10 equivalents) in the absence and presence of adenine. All samples were prepared in EMSA buffer (25 mM potassium phosphate, 75 mM KCl, 5 mM DTT, pH 7.2) and were incubated for 30 min on ice. Bands were separated on continuous gels (8% PAGE) at low powers (<500 mW, 3.5 V/cm) in TAM buffer (40 mM Tris, 20 mM acetic acid, 5 mM MgCl_2_, pH 8.3). The RNA was visualized upon staining with GelRed^TM^ (Biotium) and subsequent excitation of the bands at 254 nm.

### Native composite gel

For the analysis of ribosome stability and electrophoretic mobility shift assays, native composite gels were performed which allow the migration of native ribosomes into the gel^35^. Optimized composite gels were composed of 1.76% acrylamide and 0.68% agarose. Therefore, 13.5 mL of 0.92% agarose solution was cooled to 65 °C. Subsequently, 3 mL of prewarmed buffer (0.2 M Tris, 0.125 M acetic acid, 35% (w/v) sucrose) were added to the agarose solution. Then, 1 mL of acrylamide (29:1 acrylamide:bisacrylamide), 8.5 µL of Triton-X100 and MgCl_2_ (5 mM) was added and heated to 65 °C. Polymerization was induced by adding 100 µL of APS and 20 µL of TEMED and poured immediately into a prewarmed gel chamber. The gel was polymerized for 1 h at 4 °C. A pre-run was conducted at 200 V for 30 min under water cooling. The running buffer (40 mM Tris, 25 mM acetic acid, 5 mM MgCl_2_) was exchanged.

The 127 nt *add* riboswitch was prepared by the ligation of a 97 nt fragment (G14–G110) with the Cy5-labeled 30 nt RNA (A111-G140) (Dharmacon) as described before^36^. The RNA was pre-incubated with 1.4 equivalents of adenine. Ribosome concentration was increased from 1 to 10 equivalents at a constant RNA concentration of 1.28 µM. All samples were prepared in NMR buffer (25 mM potassium phosphate, 150 mM KCl, 5 mM MgCl_2_, pH 7.2) and a final glycerol concentration of 10% as loading buffer. Samples were loaded and the gel was run for 1 hour and 20 min at 200 V under water cooling. Bands were visualized by UV shadowing, fluorescence scan and further stained with Coomassie.

### Ribosomal RNA extraction

rRNA of 30S ribosomes were analyzed by agarose gel electrophoresis. Therefore, rRNA was extracted with 1 vol. eq. PCI. Aqueous phase was mixed with gel loading dye and analyzed by 1% agarose gel electrophoresis. The RNA was visualized upon staining with GelRed^TM^ (Biotium).

### NMR Spectroscopy

All experiments were performed at 25 °C in NMR buffer (25 mM potassium phosphate, pH 7.2, 150 mM KCl, 5 mM MgCl_2_) and 5% to 10% D_2_O. For protein-RNA studies, 5 mM DTT was added. For ligand bound RNA, 1.4 equivalents of ^13^C-^15^N-labeled adenine was added. ^13^C-^15^N-labeled adenine was synthesized as described^37^.

All spectra were referenced to DSS (4,4-dimethyl-4-silapentane-1-sulfonic acid). Nitrogen-15 and Carbon-13 chemical shifts were indirectly referenced using the ratio of the gyromagnetic ratios of proton to ^15^N (0.101329118) and ^13^C (0.251449530), respectively^38^.

For titration experiments of the ^15^N-labeled 127 nucleotide *add* adenine-sensing riboswitch with the ribosomal S1 protein, the RNA concentration was set to 40 µM. The unlabeled protein rS1 was stepwise added to the RNA in ratios ranging from 0 to 5.

For the investigation of mRNA-ribosome complexes with the 127 nucleotide ASW, samples were prepared with a concentration of 35 µM ribosomes and RNA, since higher concentrations lead to aggregation of the ribosomes.

NMR experiments were performed on an 800 MHz, 900 MHz or 950 MHz NMR spectrometer equipped with a 5 mm, z-axis gradient ^1^H {^13^C, ^15^N} TCI cryogenic probe.

BEST-TROSY (Band‐selective Excitation Short‐Transient - Transverse relaxation-optimized spectroscopy) experiments were recorded to observe ^1^H, ^15^N correlations of ^15^N isotope labeled RNA^39^. Spectra were recorded using a modified pulse program^40^.

NMR Experiments were analyzed using Bruker Biospin software TopSpin 3.5. Assignments were performed using the software Sparky 3.114^41^.

### Microscale thermophoresis

The 127 nt add riboswitch was prepared by the ligation of a 97 nt fragment (G14–G110) with the Cy5-labeled 30 nt RNA (A111–G140) (Dharmacon) as described before^36^. 30SΔS1 ribosomes were labeled with Monolith NT Protein Labeling kit red-maleimide according to the protocol (NanoTemper Technologies). The Cy5-labeled 127 nt riboswitch (30 nM) was incubated with a 1:1 dilution series of 30S ribosomes (36 µM) and 30SΔS1 ribosomes (31 µM) in the absence and presence of adenine (500 µM). Labeled 30SΔS1 (30 nM) was incubated with a 1:1 dilution series of rS1 (3 µM). The complexes were incubated for 30 min at 25 °C in NMR buffer (25 mM potassium phosphate, pH 7.2, 150 mM KCl, 5 mM MgCl_2_, 5 mM DTT) supplemented with 0.4 mg/mL BSA (New England Biolabs), 0.05% Tween20 and RNasin (Promega, Germany). The reactions were transferred to hydrophobic capillaries and measured with a microscale thermophoresis (MST) device (NanoTemper Monolith NT.115, Germany). After a primary capillary scan, the thermophoresis experiments were carried out at 25 °C with an excitation and MST power of 40% each. Four scans were recorded for each sample. The analysis was performed using MO.Affinity Analysis and the evaluation strategy T-Jump.

### In vivo assay of adenine-sensing riboswitch regulated gene expression

BL21(DE3) *E. coli* cells were transformed with pUC19 plasmid containing T7 regulatory elements and shifted green fluorescent protein (sGFP) gene under the translational control of the adenine-sensing riboswitch ASW14–122. For a pre-culture, a single colony was picked and added to 5 mL M9 minimal medium containing 100 mg/mL ampicillin and IPTG and incubated in a thermoshaker (37 °C, 120 rpm) overnight. The pre-culture was used to inoculate 50 mL M9/ampicillin main cultures with varying adenine and IPTG concentrations in biological triplicates with a start OD_600_ of 0.1, which was grown in a thermoshaker (37 °C, 120 r.p.m.). The production of sGFP was monitored via fluorescence emission (*λ*_ex_ = 484 nm and *λ*_em_ = 510 nm) with a Tecan Infinite M200 pro microplate reader (Tecan, Männedorf, Switzerland). For error estimation, three biological independent samples were measured in triplicates. Results were normalized to the growth condition of 0 mM adenine and 0 mM IPTG. As additional control, untransformed BL21(DE3) *E. coli* cells were measured.

### Coupled in vitro transcription-translation assay

The coupled in vitro transcription-translation assays and the cell extract preparation were performed as previously described^42^ to monitor protein expression levels of shifted green fluorescent protein (sGFP). The cell-free protein expression reactions were performed with in-house made S100 cell extracts from *E. coli* strain BL21 Star^TM^(DE3) and rS1-depleted ribosomes (final concentration 0.46 µM). The S100 extract preparation was adapted from the S30 extract protocol by Schwarz et al.^43^ Our preparation differs at the dialysis step. Here, the S100 extract was dialyzed at 4 °C against dialysis buffer (10 mM Tris/acetate (pH 8.2), 14 mM Mg(OAc)_2_, 60 mM KOAc, 0.5 mM DTT) using a dialysis membrane (MWCO 12-14 kDa, ZelluTrans, Carl Roth, Germany). The dialysis was performed for 3 h and the buffer was exchanged every hour. After dialysis the S100 extract was centrifuged at 40000 rpm and 4 °C for 5 h under vacuum (Beckman Coulter Optima^TM^ L-90 K). The protein is synthesized from a pUC19 plasmid containing T7 regulatory elements. The sGFP gene is under the translational control of the adenine-sensing riboswitch ASW14–122 that contains a weak ribosome-binding site and the start codon AUG. The 30 µL reactions were performed as triplicates in µClear® 384-well flat bottom microplates (Greiner Bio-One, Frickenhausen, Germany) at 25 °C for 2 h. The production of sGFP was monitored via sGFP fluorescence (*λ*_ex_ = 470 nm and *λ*_em_ = 515 nm) with a Tecan Spark® microplate reader (Tecan, Männedorf, Switzerland). For error estimation, one biological sample was measured in triplicates. Native 70S ribosomes were used as positive control.

### Structural modeling of mRNA-ribosome complexes

The riboswitch structure was modeled into the mRNA decoding channel of the 30S ribosome.

For the structural modeling, the 30S ribosomal pre-initiation complex structure with the PDB ID 5lmn was chosen. The proteins IF1, IF3 and Thx were removed from the structure.

The adenine-sensing riboswitch structure was built as follows: the holo aptamer structure (C13–G83) with the PDB ID 1y26 was used as starting point. Here, the nucleotides C13 and G83 were removed from the structure.

To build the single stranded region of the mRNA, the online tool RNAthread (ROSIE) was used^44,45^. Here, the single stranded mRNA from the 5lmn structure with 20 nucleotides was used as input structure. The nucleotides were replaced in steps of 10–20 nucleotides with the desired ASW sequence. The resulting structure was then renumbered accordingly to the ASW sequence using the renumber_PDB tool.

Sequences were aligned in bins of two, each containing two overlapping nucleotides. The alignment was performed in Pymol. This procedure ensured preservation of the correct backbone geometry. The redundant nucleotides from the alignment were removed manually in text editor. The mRNA in the 5lmn structure was replaced by the modeled ASW structure, maintaining the same position of the GAG triplet (G110–G112) from the Shine–Dalgarno sequence, that base pairs with the rRNA (C1536–C1538).

### Reporting summary

Further information on research design is available in the Nature Research Reporting Summary linked to this article.

## Supplementary information

Supplementary Information

Peer Review File

Description of Additional Supplementary Files

Supplementary Data 1

Reporting Summary

## Data Availability

The data needed to evaluate the conclusions in the paper are present in the paper and/or the supplementary information, and are available from the corresponding author upon reasonable request. The structural model that was generated in this study based on the publically available structures 1Y26 and 5MLN is provided in Supplementary Data 1. Source data are provided with this paper.

## Source data

Source Data

## References

[1] Shine J, Dalgarno L (1974). The 3′-terminal sequence of *Escherichia coli* 16S ribosomal RNA: complementarity to nonsense triplets and ribosome binding sites. Proc. Natl Acad. Sci. USA.

[2] Steitz JA, Jakes K (1975). How ribosomes select initiator regions in mRNA: base pair formation between the 3′ terminus of 16S rRNA and the mRNA during initiation of protein synthesis in *Escherichia coli*. Proc. Natl Acad. Sci. USA.

[3] Marzi S (2007). Structured mRNAs regulate translation initiation by binding to the platform of the ribosome. Cell.

[4] De Smit MH, Van Duin J (2003). Translational standby sites: how ribosomes may deal with the rapid folding kinetics of mRNA. J. Mol. Biol..

[5] Romilly C, Deindl S, Wagner EGH (2019). The ribosomal protein S1-dependent standby site in tisB mRNA consists of a single-stranded region and a 5′ structure element. Proc. Natl Acad. Sci. USA.

[6] Studer SM, Joseph S (2006). Unfolding of mRNA secondary structure by the bacterial translation initiation complex. Mol. Cell.

[7] Darfeuille F, Unoson C, Vogel J, Wagner EGH (2007). An antisense RNA inhibits translation by competing with standby ribosomes. Mol. Cell.

[8] Simonetti A (2009). A structural view of translation initiation in bacteria. Cell. Mol. Life Sci..

[9] Breaker, R. R. Riboswitches and translation control. *Cold Spring Harb. Perspect. Biol*. **10**, 1–14 (2018). 10.1101/cshperspect.a032797 PMC6211393 29844057

[10] Rinaldi AJ, Lund PE, Blanco MR, Walter NG (2016). The Shine-Dalgarno sequence of riboswitch-regulated single mRNAs shows ligand-dependent accessibility bursts. Nat. Commun..

[11] Bédard ASV, Hien EDM, Lafontaine DA (2020). Riboswitch regulation mechanisms: RNA, metabolites and regulatory proteins. Biochim. Biophys. Acta.

[12] Reining A (2013). Three-state mechanism couples ligand and temperature sensing in riboswitches. Nature.

[13] Warhaut S (2017). Ligand-modulated folding of the full-length adenine riboswitch probed by NMR and single-molecule FRET spectroscopy. Nucleic Acids Res..

[14] Lemay, J. F. et al. Comparative study between transcriptionally- and translationally-acting adenine riboswitches reveals key differences in riboswitch regulatory mechanisms. *PLoS Genet*. **7**, e1001278 (2011). 10.1371/journal.pgen.1001278 PMC3024265 21283784

[15] Fürtig, B. et al. Refolding through a linear transition state enables fast temperature adaptation of a translational riboswitch. *Biochemistry* **59**, 1081–1086 (2020). 10.1021/acs.biochem.9b01044 32134253

[16] Yusupova GZ, Yusupov MM, Cate JHD, Noller HF (2001). The path of messenger RNA through the ribosome. Cell.

[17] Coetzee T, Herschlag D, Belfort M (1994). *Escherichia coli* proteins, including ribosomal protein S12, facilitate in vitro splicing of phage T4 introns by acting as RNA chaperones. Genes Dev..

[18] Semrad K, Green R, Schroeder R (2004). RNA chaperone activity of large ribosomal subunit proteins from *Escherichia coli*. RNA.

[19] Aseev LV, Boni IV (2011). Extraribosomal functions of bacterial ribosomal proteins. Mol. Biol..

[20] Duval M (2013). *Escherichia coli* ribosomal protein S1 unfolds structured mRNAs onto the ribosome for active translation initiation. PLoS Biol..

[21] Hajnsdorf E, Boni IV (2012). Multiple activities of RNA-binding proteins S1 and Hfq. Biochimie.

[22] Sengupta J, Agrawal RK, Frank J (2001). Visualization of protein S1 within the 30S ribosomal subunit and its interaction with messenger RNA. Proc. Natl Acad. Sci. USA.

[23] Byrgazov K (2014). Structural basis for the interaction of protein S1 with the *Escherichia coli* ribosome. Nucleic Acids Res..

[24] Wilson DN, Nierhaus KH (2005). Ribosomal proteins in the spotlight. Crit. Rev. Biochem. Mol. Biol..

[25] Qureshi, N. S., Bains, J. K., Sreeramulu, S., Schwalbe, H. & Fürtig, B. Conformational switch in the ribosomal protein S1 guides unfolding of structured RNAs for translation initiation. *Nucleic Acids Res*. **46**, 10917–10929 (2018). 10.1093/nar/gky746 PMC6237739 30124944

[26] Saguy, M. et al. Ribosomal protein S1 influences trans -translation in vitro and in vivo. *Nucleic Acid Res*. **35**, 2368–2376 (2007). 10.1093/nar/gkm100 PMC1874662 17392345

[27] Kitakawa M, Isono K (1982). An amber mutation in the gene rpsA for ribosomal protein S1 in *Escherichia coli*. MGG Mol. Gen. Genet..

[28] Lund, P. E., Chatterjee, S., Daher, M. & Walter, N. G. Protein unties the pseudoknot: S1-mediated unfolding of RNA higher order structure. *Nucleic Acid Res*. **48**, 2107–2125 (2019). 10.1093/nar/gkz1166 PMC7038950 31832686

[29] Sterk, M., Romilly, C. & Wagner, E. G. H. Unstructured 5′-tails act through ribosome standby to override inhibitory structure at ribosome binding sites. *Nucleic Acids Res*. **46**, 4188-4199 (2018). 10.1093/nar/gky073 PMC5934652 29420821

[30] Beckert B (2018). Structure of a hibernating 100S ribosome reveals an inactive conformation of the ribosomal protein S1. Nat. Microbiol..

[31] Fürtig B, Richter C, Wöhnert J, Schwalbe H (2003). NMR spectroscopy of RNA. ChemBioChem.

[32] Bains JK (2019). Combined smFRET and NMR analysis of riboswitch structural dynamics. Methods.

[33] Ederth J, Mandava CS, Dasgupta S, Sanyal S (2009). A single-step method for purification of active His-tagged ribosomes from a genetically engineered Escherichia coli. Nucleic Acids Res..

[34] Christodoulou J (2004). Heteronuclear NMR investigations of dynamic regions of intact *Escherichia coli* ribosomes. Proc. Natl Acad. Sci. USA.

[35] Nadano, D., Aoki, C., Yoshinaka, T., Irie, S. & Sato, T. A. Electrophoretic characterization of ribosomal subunits and proteins in apoptosis: specific downregulation of S11 in staurosporine-treated human breast carcinoma cells. *Biochemistry* **40**, 15184–15193 (2001). 10.1021/bi0108397 11735401

[36] Duss, O., Maris, C., Von Schroetter, C. & Allain, F. H. T. A fast, efficient and sequence-independent method for flexible multiple segmental isotope labeling of RNA using ribozyme and RNase H cleavage. *Nucleic Acids Res*. **38**, e188 (2010). 10.1093/nar/gkq756 PMC2978384 20798173

[37] Noeske J (2005). An intermolecular base triple as the basis of ligand specificity and affinity in the guanine- and adenine-sensing riboswitch RNAs. Proc. Natl Acad. Sci. USA.

[38] Wishart DS (1995). 1H, 13C and 15N chemical shift referencing in biomolecular NMR. J. Biomol. NMR.

[39] Solyom Z (2013). BEST-TROSY experiments for time-efficient sequential resonance assignment of large disordered proteins. J. Biomol. NMR.

[40] Favier A, Brutscher B (2011). Recovering lost magnetization: polarization enhancement in biomolecular NMR. J. Biomol. NMR.

[41] Lee W, Tonelli M, Markley JL (2015). NMRFAM-SPARKY: enhanced software for biomolecular NMR spectroscopy. Bioinformatics.

[42] Kaldenhoff, R., Bernhard, F., Kai, L. & Do, V. Artificial environments for the co-translational stabilization of cell-free expressed proteins. *PLoS ONE* **8**, e56637 (2013). 10.1371/journal.pone.0056637 PMC3579822 23451062

[43] Schwarz D (2007). Preparative scale expression of membrane proteins in Escherichia coli-based continuous exchange cell-free systems. Nat. Protoc..

[44] Watkins AM, Rangan R, Das R (2020). FARFAR2: improved de novo rosetta prediction of complex global RNA folds. Structure.

[45] Lyskov S (2013). Serverification of molecular modeling applications: the rosetta online server that includes everyone (ROSIE). PLoS ONE.

