# Supplementary material for: Switching at the ribosome: riboswitches need rProteins as modulators to regulate translation
Source: Nat Commun. 2021 Aug 5;12:4723. doi: 10.1038/s41467-021-25024-5 (PMC8342710; doi:10.1038/s41467-021-25024-5)
Supplement: Supplementary file 1 — Supplementary Information [file 41467_2021_25024_MOESM1_ESM.pdf]

Supplementary Materials for

Switching at the Ribosome: Riboswitches need rProteins as modulators to regulate translation

Vanessa de Jesus[1], Nusrat S. Qureshi[1], Sven Warhaut[1], Jasleen K. Bains[1], Marina S. Dietz[2], Mike Heilemann[2], Harald Schwalbe[1], Boris Fürtig[1*]

Correspondence to: fuertig@nmr.uni-frankfurt.de

This PDF file includes:

Supplementary Figure 1 to 11

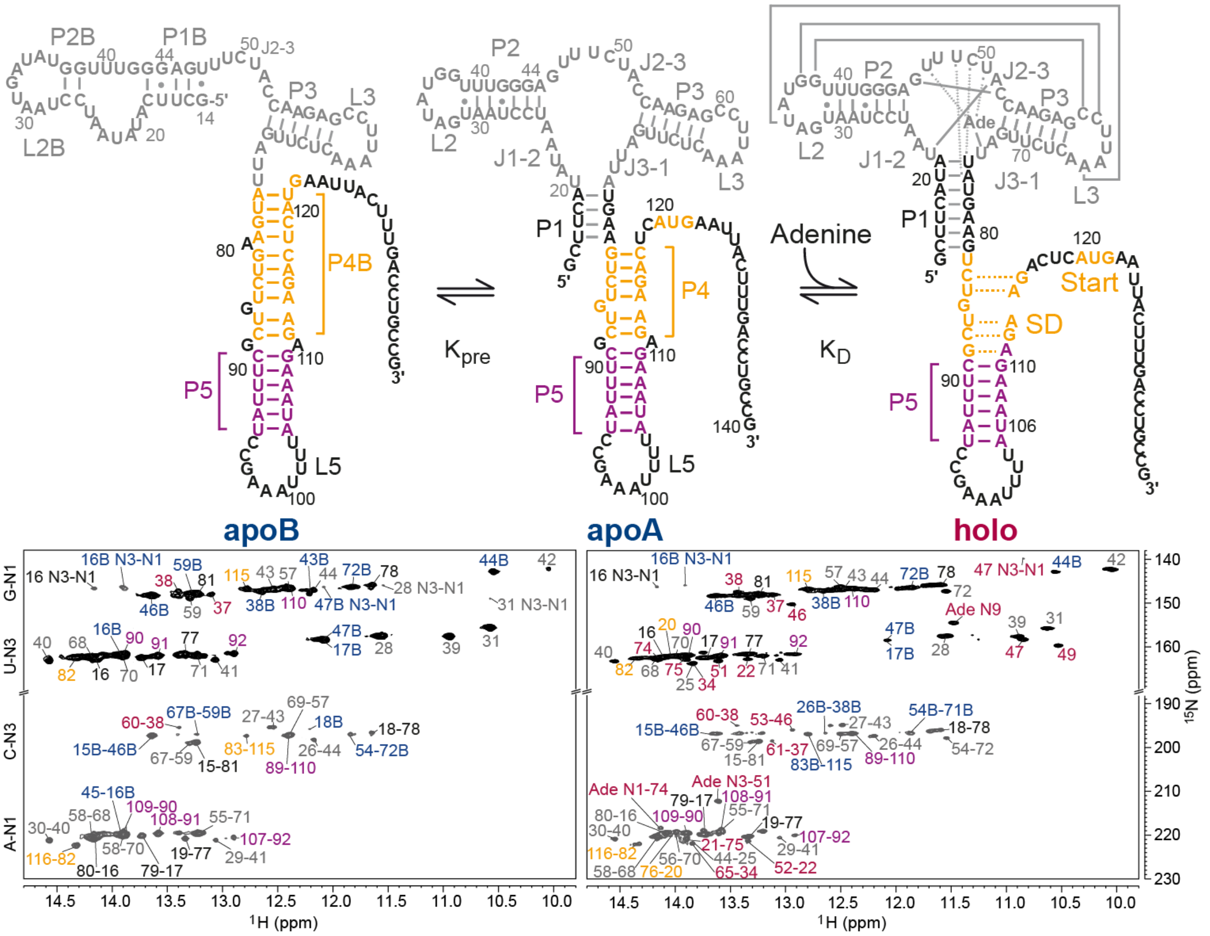

Supplementary Figure 1: 2D-[^{1}H-^{15}N]-BEST-TROSY-HNN-COSY of adenine-sensing-riboswitch in absence of adenine (apo; left) and presence of ^{13}C-^{15}N-labeled adenine (holo; right) at 25°C. The secondary structure is shown on top. Peaks are color coded according to the secondary structure elements. HNN-COSY spectra were recorded in potassium phosphate buffer (25 mM KPi, 150 mM KCl and 5 mM $MgCl_2$, pH 7.2) on a 800 MHz NMR spectrometer at 25°C with HNN transfer of 30 ms.

HNN-COSY spectrum of ASW apo was recorded with an RNA concentration of 465 µM. The interscan delay was set to 0.3 seconds and 496 scans per increment were recorded. The proton carrier frequency was set to the resonance frequency of the solvent (4.7 ppm). The spectrum was recorded with a spectral width of 24 ppm in the direct dimension and 100 ppm in the indirect dimension. The spectrum was recorded with an acquisition time of 53 ms representing 2048 points in the ^{1}H dimension. In the indirect dimension, 360 points were recorded with an acquisition time of 22 ms.

HNN-COSY spectrum of ASW holo was recorded with an RNA concentration of 229 µM with 1.4 eq. adenine. The interscan delay was set to 0.3 seconds and 512 scans per increment were recorded. The proton carrier frequency was set to the resonance frequency of the solvent (4.7 ppm). The spectrum was recorded with a spectral width of 24 ppm in the direct dimension and 100 ppm in the indirect dimension. The spectrum was recorded with an acquisition time of 79 ms representing 3074 points in the ^{1}H dimension. In the indirect dimension, 512 points were recorded with an acquisition time of 31 ms.

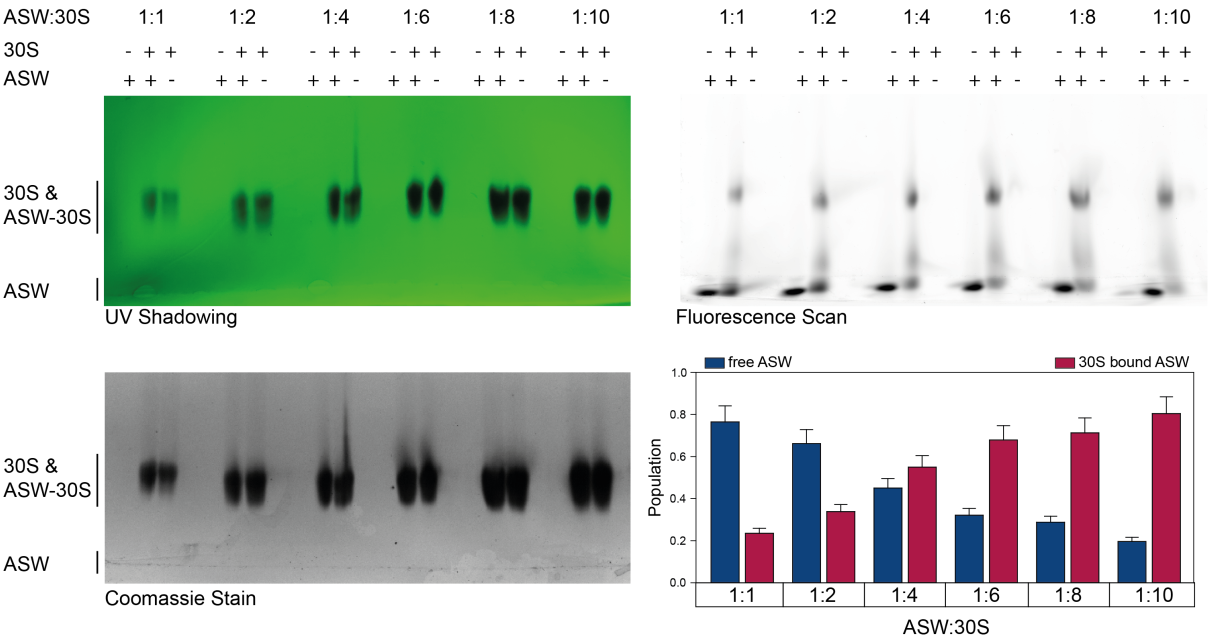

Supplementary Figure 2: Native composite gel electrophoreses of mRNA-ribosome complexes. The Cy5-labeled adenine-sensing riboswitch (ASW) was pre-incubated with 1.4 equivalents of adenine. 30S ribosome concentration was increased from 1 to 10 equivalents at a constant RNA concentration of 1.28 µM. All samples were prepared in NMR buffer (25 mM potassium phosphate, 150 mM KCl, 5 mM $MgCl_2$, pH 7.2). Bands were visualized by UV shadowing, fluorescence scan and further stained with Coomassie. Fractions of free ASW and ASW bound to 30S ribosomes are plotted for different ASW:30S ribosome ratios. The intensities were extracted from the fluorescence scan gel with Image lab software. Error bars were calculated with 10% from the standard deviation of all RNA-only samples (n=6). Source data are provided as a Source data file.

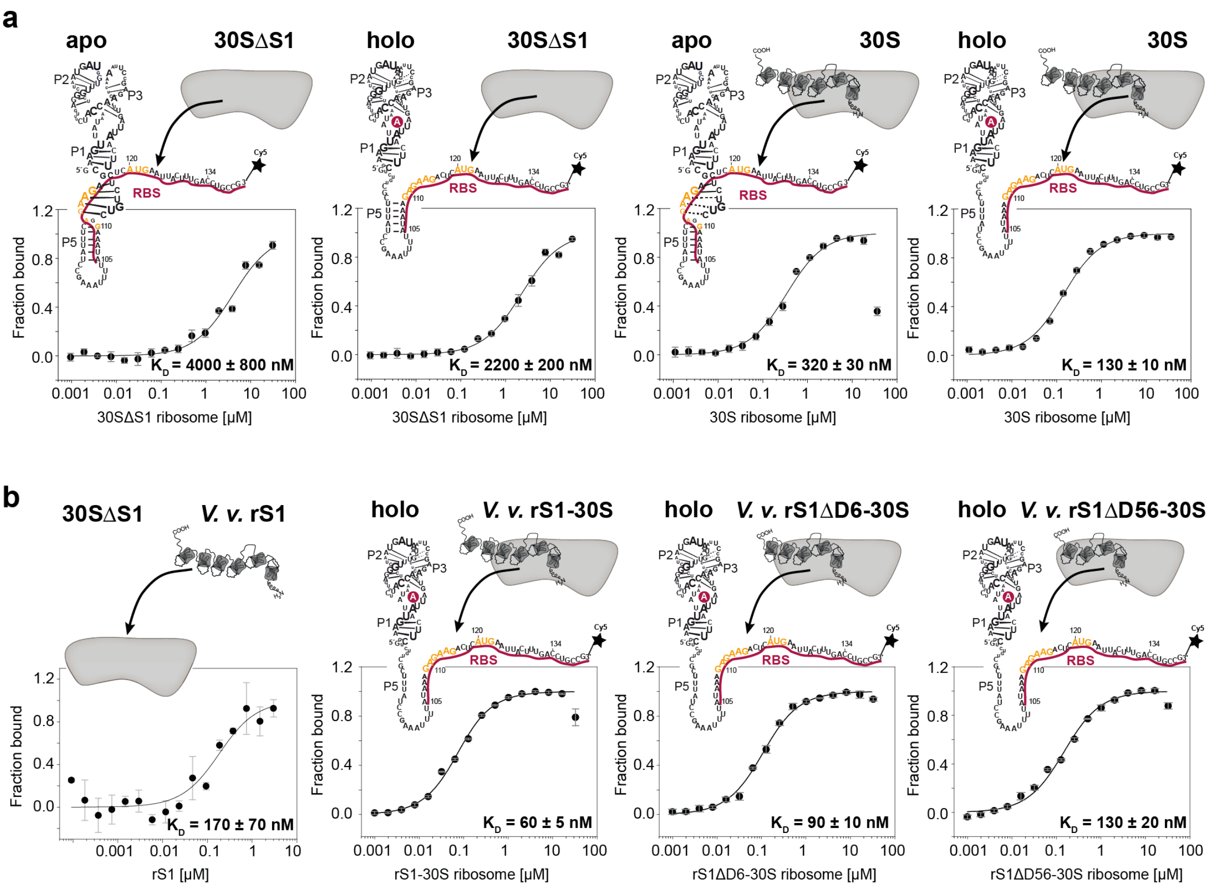

Supplementary Figure 3: Binding affinity (K_D) of mRNA-ribosome complexes by microscale thermophoresis (MST). Adenine-sensing riboswitch from *Vibrio vulnificus* (*V. v.*) was Cy5-labeled at 3'-end and measured in the absence (apo) and presence (holo) of adenine (500 µM). The complexes were incubated for 30 minutes at 25°C in NMR buffer (25 mM potassium phosphate, pH 7.2, 150 mM KCl, 5 mM $MgCl_2$, 5 mM DTT) supplemented with 0.4 mg/mL BSA (New England Biolabs), 0.05% Tween20 and RNasin (Promega, Germany). Thermophoresis experiments were carried out at 25°C. Four scans were recorded for each sample (mean ± s.d.). (a) Cy5-labeled ASW was titrated with 30SΔS1 and 30S ribosomes in absence and presence of adenine. (b) Affinity of *Vibrio vulnificus* rS1 protein to *E. coli* 30SΔS1 ribosomes. In addition, the affinity of *V.v.* rS1-30S complexes towards the ASW was determined as well as deletion mutants of *V.v.* rS1 (rSΔD6 and rSΔD56, without domain 6 and 56, respectively). Source data are provided as a Source data file.

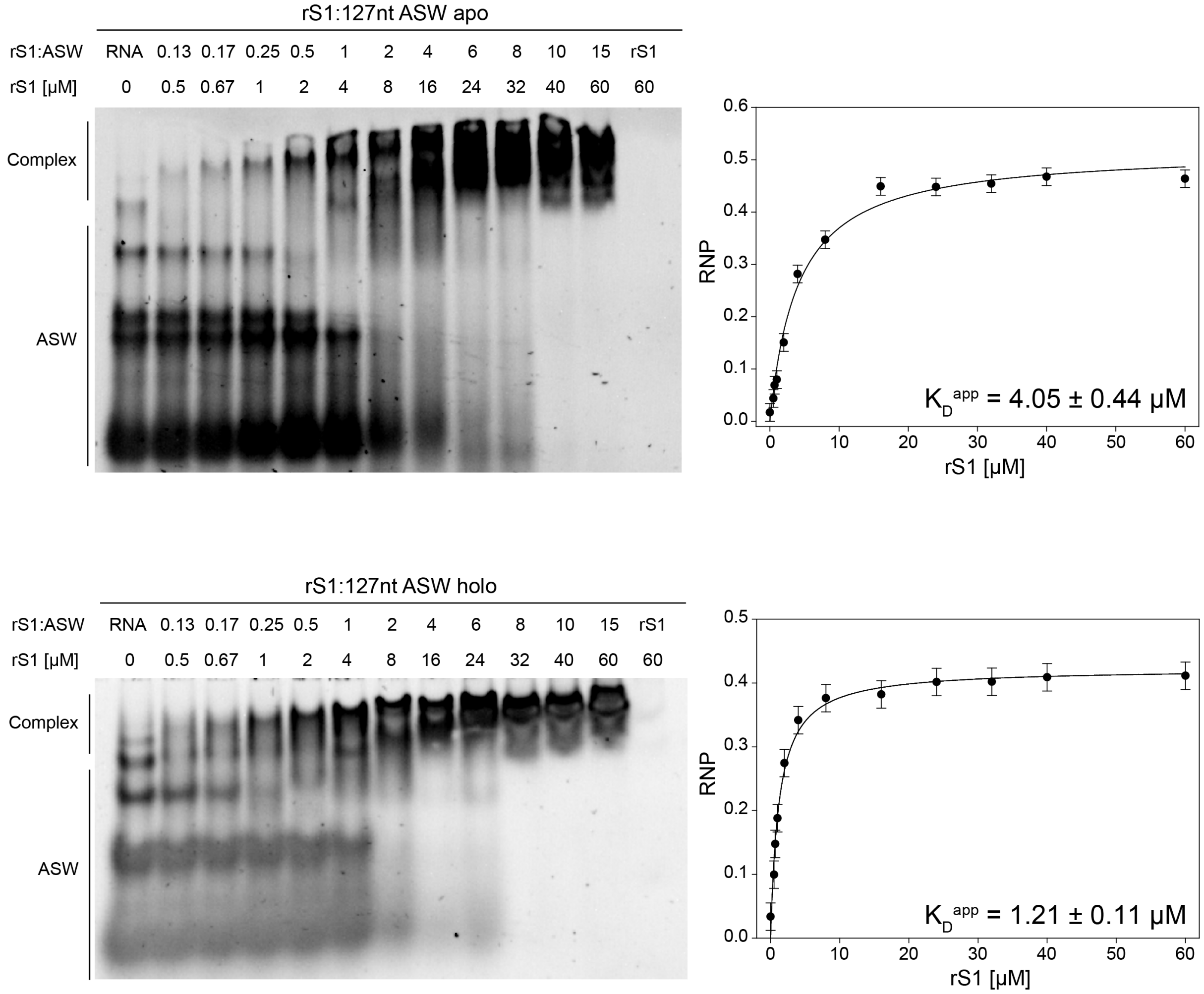

Supplementary Figure 4: Electrophoretic mobility shift assays (EMSAs) of rS1 with the 127mer adenine-sensing riboswitch (ASW) in TAM buffer. EMSAs were performed in the absence (apo) and presence (holo) of adenine. EMSAs were performed with constant concentration of ASW (4 µM). Increasing concentration of rS1 are indicated on top of the gels (0-15 equivalents). Upper bands correspond to protein-RNA complexes (RNP, ribonucleoprotein complexes), lower bands correspond to free RNA. 20 pmol of sample were applied per lane. On the right, RNP population is plotted against rS1 concentration. The RNP intensities were extracted from the gel with Image lab software and normalized to free RNA intensity. Error was calculated by integration of the noise over the same size of pixels at same height. Apparent dissociation constants K_D^{app} are given in the plots. Source data are provided as a Source data file.

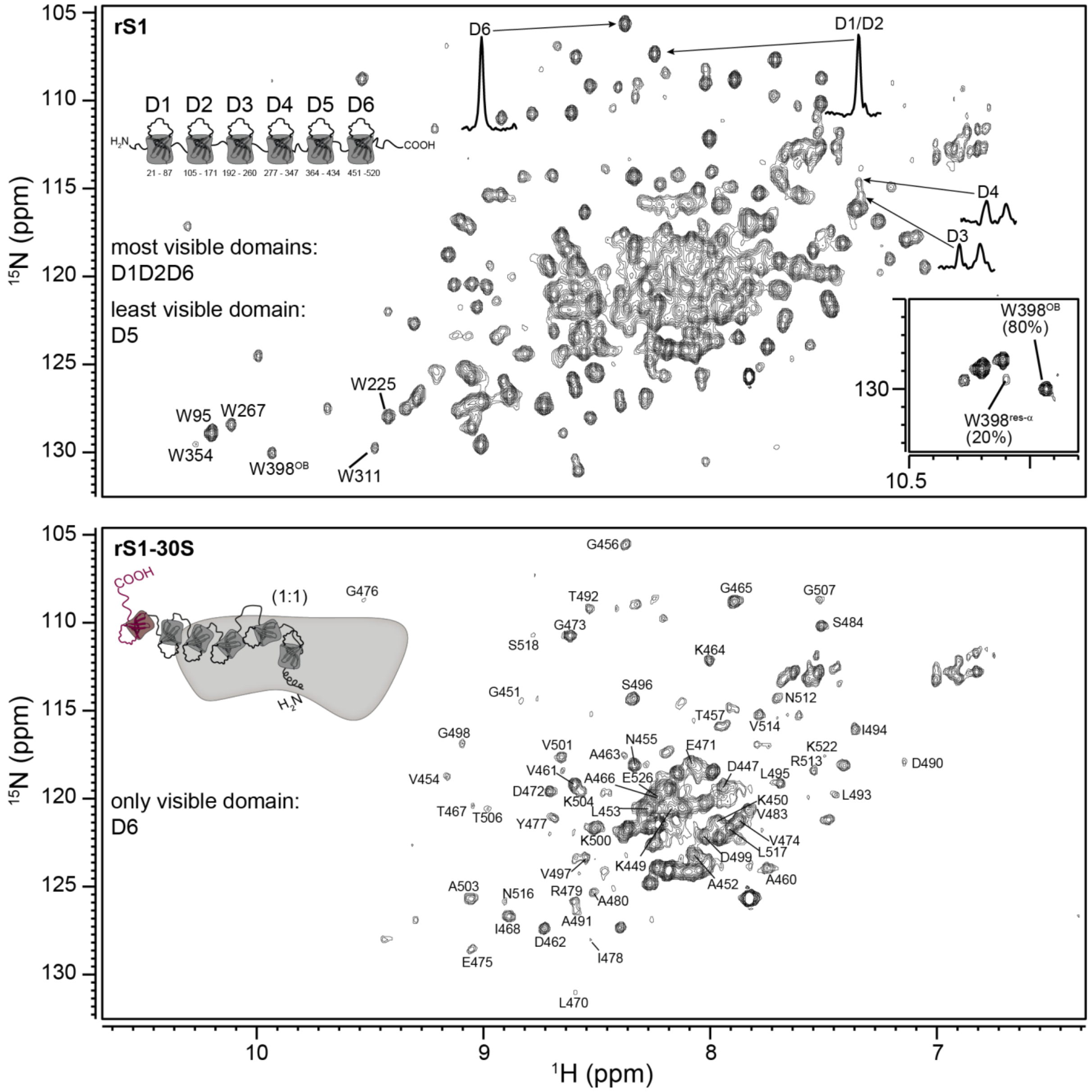

Supplementary Figure 5: Interaction of *Vibrio vulnificus* rS1 with 30S ribosomes. [^{1}H-^{15}N]-BEST-TROSY experiments of ^{15}N-labeled rS1 (30 µM) and rS1-30S were recorded in NMR buffer (25 mM KPi, 150 mM KCl, 5 mM $MgCl_2$, 5 mM DTT, pH 7.2) on a 900 MHz NMR spectrometer at 35°C. The upper spectrum shows the rS1-only sample. The lower spectrum displays ^{15}N-labeled rS1 in presence of one equivalent unlabeled rS1-depleted 30S ribosomes. The complex is represented, and the only visible parts are highlighted in pink (D6). The assignment of domain D6 is annotated. Unassigned peaks correspond to either unassigned D6 peaks or to C-terminal tail.

a

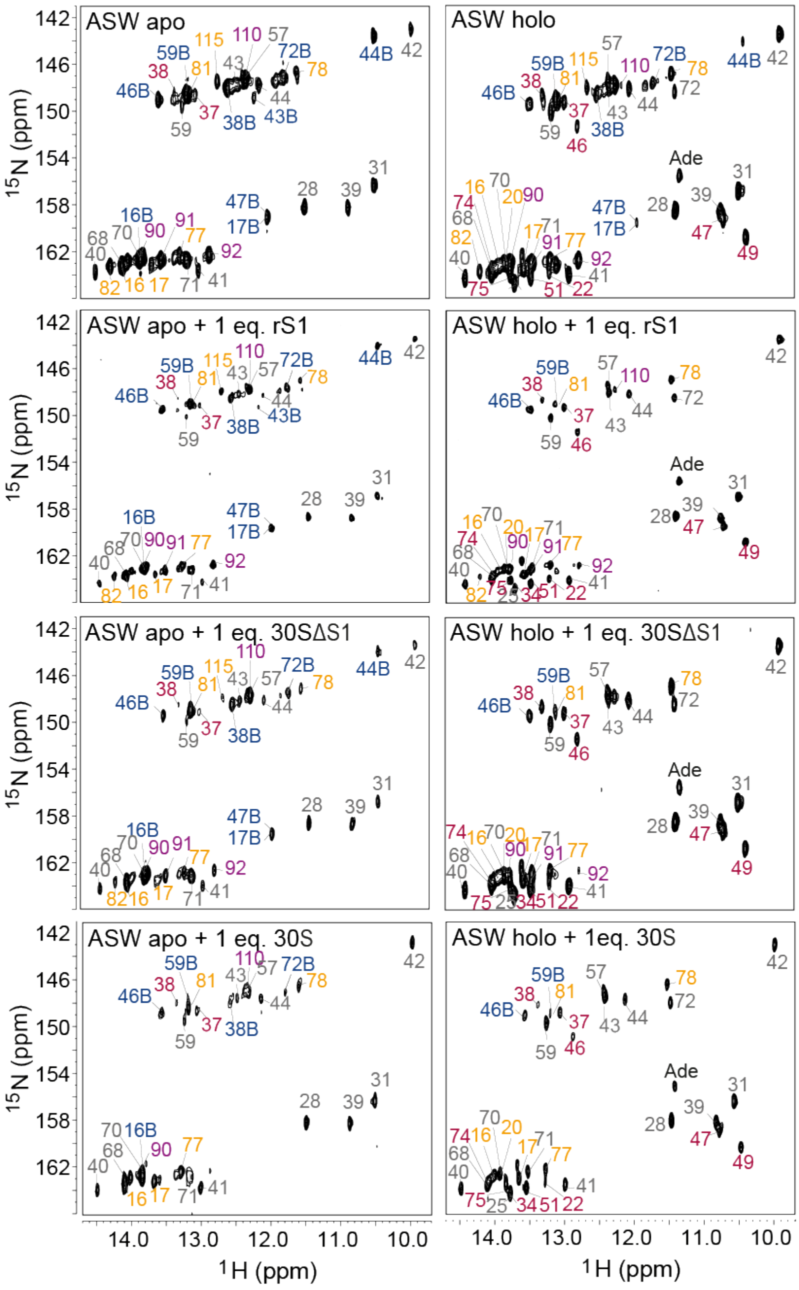
ASW apo
ASW holo
ASW apo + 1 eq. rS1
ASW holo + 1 eq. rS1
ASW apo + 1 eq. 30SΔS1
ASW holo + 1 eq. 30SΔS1
ASW apo + 1 eq. 30S
ASW holo + 1eq. 30S
15N (ppm)
142
146
150
154
158
162
14.0
13.0
12.0
11.0
10.0
1H (ppm)

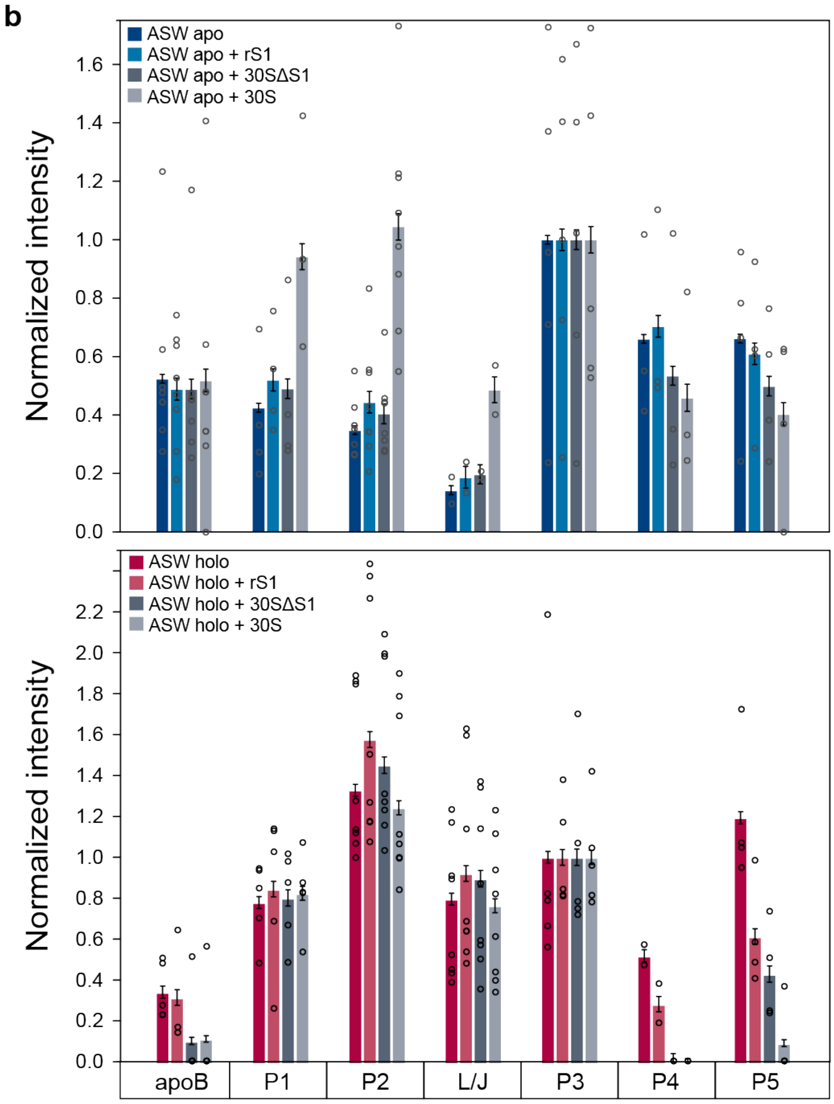

Supplementary Figure 6: NMR spectroscopic investigations of mRNA-ribosome complexes. (a) [^{1}H-^{15}N]-BEST-TROSY experiments of ASW, ASW-rS1, ASW-30SΔS1 and ASW-30S in absence and presence of ^{13}C-^{15}N-labeled adenine. BEST-TROSY spectra were recorded in NMR buffer (25 mM KPi, 150 mM KCl, 5 mM $MgCl_2$, 5 mM DTT, pH 7.2) on a 800 MHz NMR spectrometer at 25°C. BEST-TROSY spectra were recorded with an RNA concentration of 35 µM for ASW and ASW-30S and 40 µM for ASW-rS1 complexes. For ASW, ASW-30SΔS1 and ASW-30S, spectra were recorded with the following parameters: The interscan delay was set to 0.2 seconds and 4672 scans per increment were recorded. The proton carrier frequency was set to the resonance frequency of the solvent (4.7 ppm). The spectra were recorded with a spectral width of 24 ppm in the direct dimension and 30 ppm in the indirect dimension. The spectra were recorded with an acquisition time of 53 ms representing 2048 points in the ^{1}H dimension. In the indirect dimension, 60 points were recorded with an acquisition time of 12 ms. For ASW-rS1, the interscan delay was set to 0.2 seconds and 1120 scans per increment were recorded. The proton carrier frequency was set to the resonance frequency of the solvent (4.7 ppm). The spectra were recorded with a spectral width of 24 ppm in the direct dimension and 30 ppm in the indirect dimension. The spectra were recorded with an acquisition time of 53 ms representing 2048 points in the ^{1}H dimension. In the indirect dimension, 128 points were recorded with an acquisition time of 26 ms. Spectra are identical to those presented in Figure 3. (b) Normalized intensities of peaks according to the secondary structure elements. Peaks were normalized to helix P3. Source data are provided as a Source data file. Error bars represent the standard deviation of the average weighted by the signal-to-noise ratio of the individual peaks.

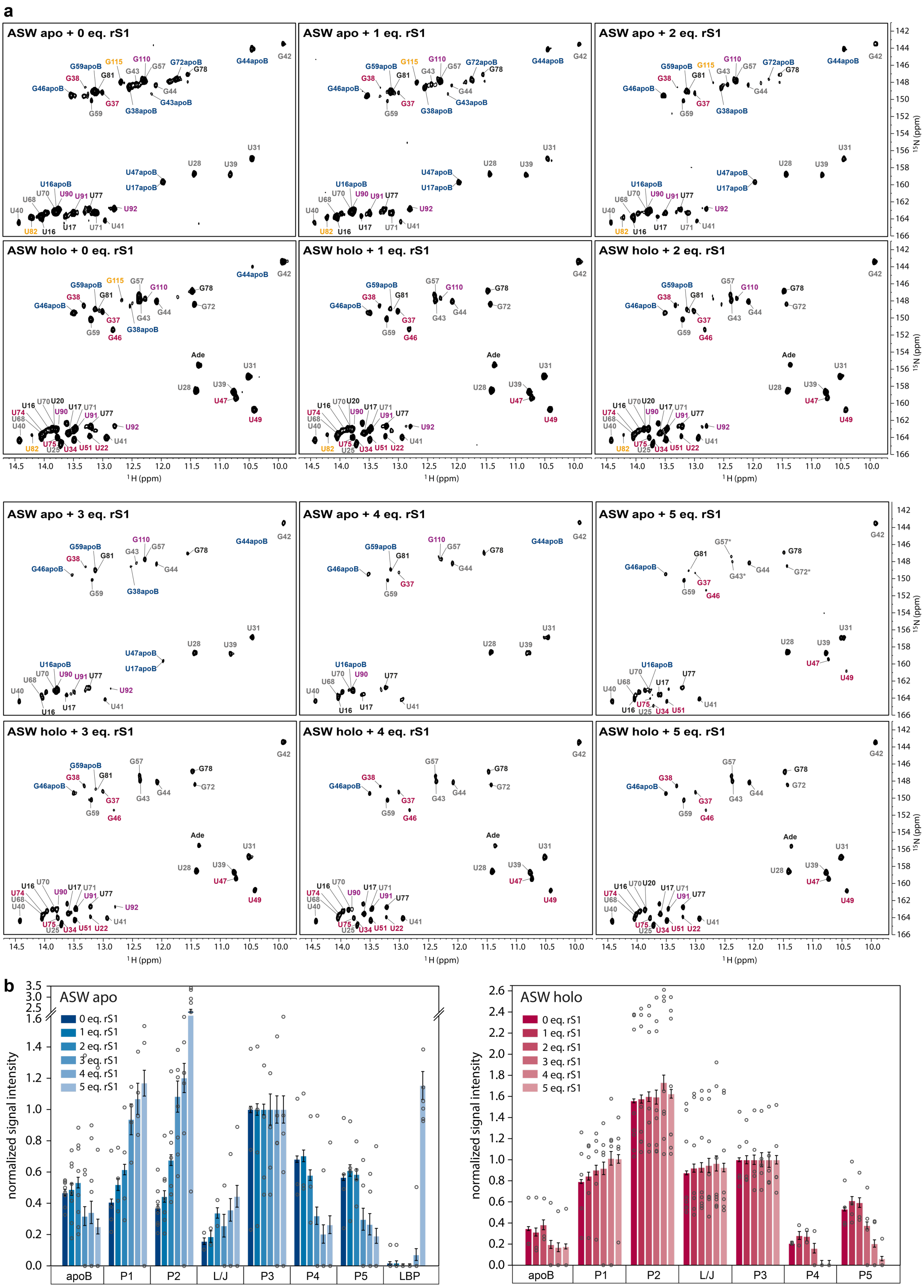

Supplementary Figure 7: [^{1}H-^{15}N]-BEST-TROSY NMR experiments of the titration of adenine-sensing riboswitch (ASW) from *Vibrio vulnificus* with molar ratios of *Vibrio vulnificus* rS1 protein. (a) BEST-TROSY spectra were recorded in NMR buffer (25 mM KPi, 150 mM KCl, 5 mM $MgCl_2$, 5 mM DTT, pH 7.2) on a 800 MHz NMR spectrometer at 25°C. BEST-TROSY spectra were recorded with an RNA concentration of 40 µM. The interscan delay was set to 0.2 seconds and 1120 scans per increment were

recorded. The proton carrier frequency was set to the resonance frequency of the solvent (4.7 ppm). The spectra were recorded with a spectral width of 24 ppm in the direct dimension and 30 ppm in the indirect dimension. The spectra were recorded with an acquisition time of 53 ms representing 2048 points in the ^{1}H dimension. In the indirect dimension, 128 points were recorded with an acquisition time of 26 ms. (b) Normalized intensities of peaks according to the secondary structure elements (loops and junctions (L/J), ligand binding pocket (LBP)). Peaks were normalized to helix P3. Source data are provided as a Source data file. Error bars represent the standard deviation of the average weighted by the signal-to-noise ratio of the individual peaks.

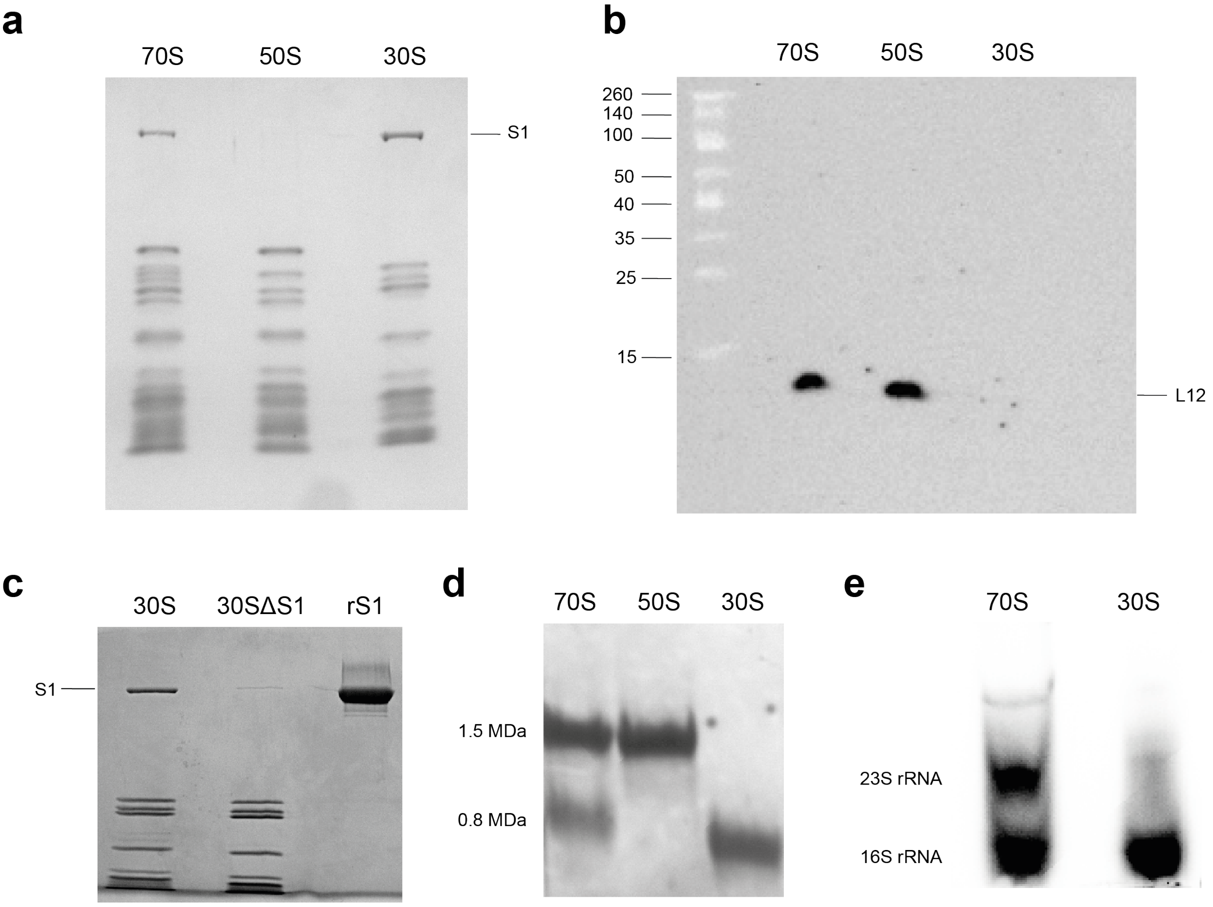

Supplementary Figure 8: Analysis of purified ribosomes, ribosomal proteins and ribosomal RNA. (a) 12% SDS-PAGE of purified 70S, 50S and 30S ribosomes. The rS1 protein is not present in the 50S ribosomal protein sample. Gel was visualized by silver staining. Source data are provided as a Source data file. (b) Western Blot analysis using 6x-His Tag Antibody for the selective detection of the modified his-tagged ribosomal protein L12. (c) Analysis of native and rS1 depleted 30S ribosomal subunits and rS1 protein. SDS-PAGE of purified 30S and 30SΔS1 ribosomes and full-length rS1 protein used in the NMR experiments. The gel was stained with Coomassie. Source data are provided as a Source data file. (d) Native gel electrophoresis of ribosomal complexes with 70S, 50S and 30S ribosomes under dissociating conditions. Source data are provided as a Source data file. (e) Analysis of rRNA composition. Gel was visualized by GelRed. The data presented here was represent examples of data repeatedly generated for each new preparation of material which always yielded the identical results.

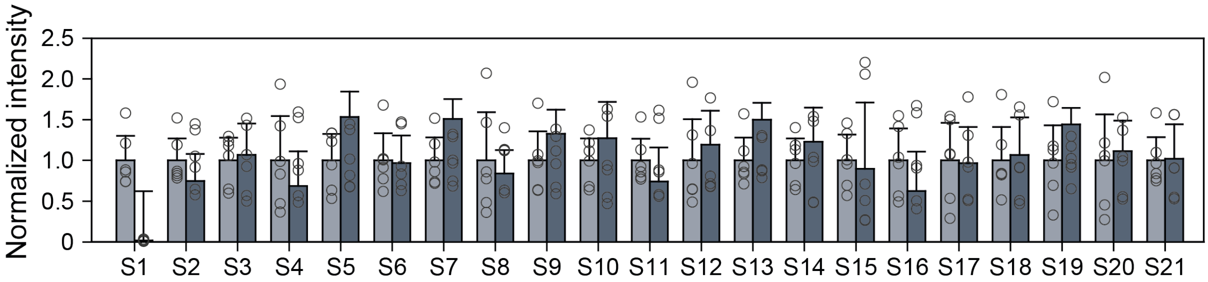

Supplementary Figure 9: Quantitative mass spectrometry analysis of 30S and 30SΔS1 ribosomes. Three biologically independent samples from different purification procedures were measured in duplicates for error estimation (mean ± s.d.). The intensities of each protein are normalized to 30S ribosomes. Source data are provided as a Source data file.

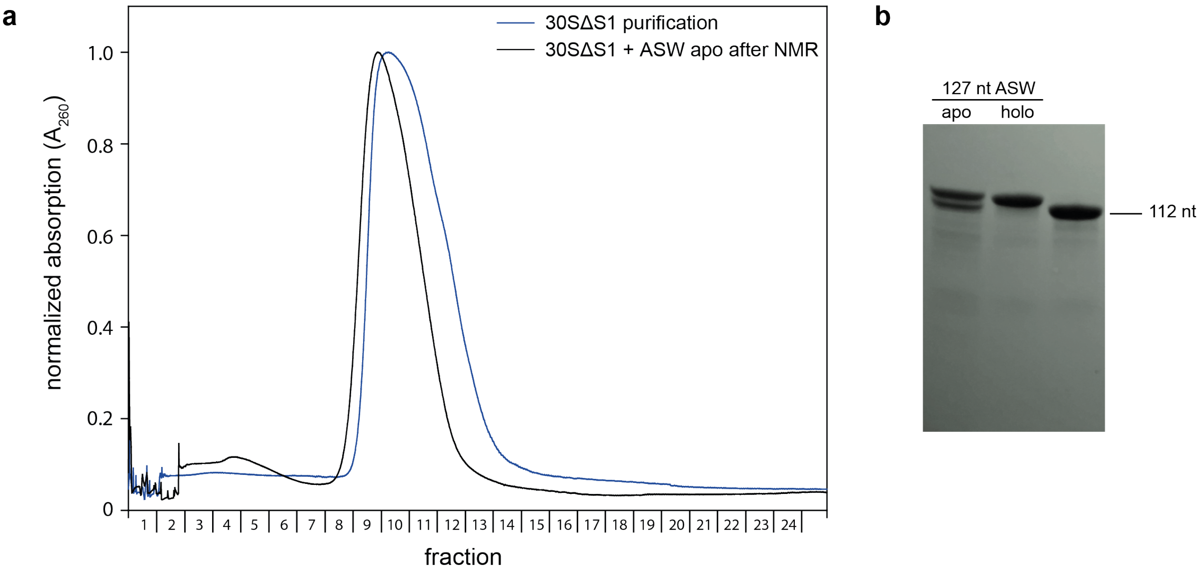

Supplementary Figure 10: Analysis of ribosomal and riboswitch integrity after NMR measurements. (a) Sucrose gradient profiles of ribosomes before and after NMR measurements, here shown exemplary for 30SΔS1 particles. For analysis, 1/4 of NMR sample was applied to sucrose gradient. (b) Denaturing PAGE of NMR samples after NMR measurements to monitor RNA integrity. The 127mer ASW were measured in complex with the rS1 protein (exemplary shown). 112 nt RNA was used as reference. Source data are provided as a Source data file.

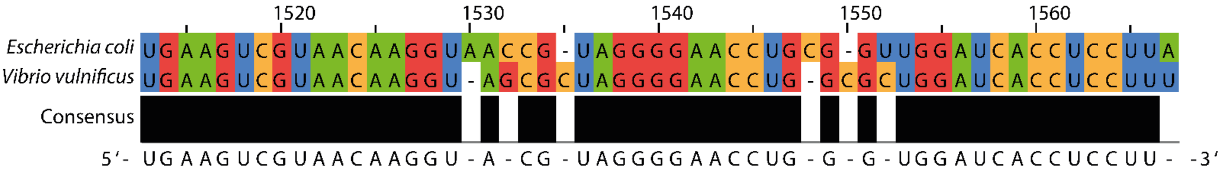

Supplementary Figure 11: Comparison of the 3'-end of 16S rRNAs of *E. coli* with *Vibrio vulnificus*. The last nucleotides of the rRNA harbor the anti-Shine-Dalgarno sequence. As the cognate ribosome from *V. vulnificus* is not accessible for practical reasons, the interaction is studied here with the homologous *E. coli* ribosome. The 3'-end of the 16S rRNA in *E. coli* which harbors the aSD sequence is nearly identical (identity = 98%) to that of *Vibrio vulnificus* (Supplementary Figure 4) supporting the validity of using *E. coli* ribosomes to study the molecular basis of translation initiation for the *add* riboswitch from *V. vulnificus*. Sequence identity was compared with Jalview.
