# Supplementary material for: Switching at the ribosome: riboswitches need rProteins as modulators to regulate translation
Source: Nat Commun. 2021 Aug 5;12:4723. doi: 10.1038/s41467-021-25024-5 (PMC8342710; doi:10.1038/s41467-021-25024-5)
Supplement: Supplementary file 3 — Description of Additional Supplementary Files [file 41467_2021_25024_MOESM3_ESM.pdf]

Description of Additional Supplementary Files

File Name: Supplementary Data 1
Description: Modelled Structure of 30S bound adenine sensing riboswitch in ligand bound conformation
