# Supplementary material for: Switching at the ribosome: riboswitches need rProteins as modulators to regulate translation
Source: Nat Commun. 2021 Aug 5;12:4723. doi: 10.1038/s41467-021-25024-5 (PMC8342710; doi:10.1038/s41467-021-25024-5)
Supplement: Supplementary file 5 — Reporting Summary [file 41467_2021_25024_MOESM5_ESM.pdf]

nature portfolio

Corresponding author(s): Boris Fürtig

Last updated by author(s): Jul 9, 2021

Reporting Summary

Nature Portfolio wishes to improve the reproducibility of the work that we publish. This form provides structure for consistency and transparency in reporting. For further information on Nature Portfolio policies, see our Editorial Policies and the Editorial Policy Checklist.

Statistics

For all statistical analyses, confirm that the following items are present in the figure legend, table legend, main text, or Methods section.

n/a	Confirmed	
☐	☒	The exact sample size (n) for each experimental group/condition, given as a discrete number and unit of measurement
☐	☒	A statement on whether measurements were taken from distinct samples or whether the same sample was measured repeatedly
☒	☐	The statistical test(s) used AND whether they are one- or two-sided<br>*Only common tests should be described solely by name; describe more complex techniques in the Methods section.*
☒	☐	A description of all covariates tested
☒	☐	A description of any assumptions or corrections, such as tests of normality and adjustment for multiple comparisons
☐	☒	A full description of the statistical parameters including central tendency (e.g. means) or other basic estimates (e.g. regression coefficient) AND variation (e.g. standard deviation) or associated estimates of uncertainty (e.g. confidence intervals)
☒	☐	For null hypothesis testing, the test statistic (e.g. F, t, r) with confidence intervals, effect sizes, degrees of freedom and P value noted<br>*Give P values as exact values whenever suitable.*
☒	☐	For Bayesian analysis, information on the choice of priors and Markov chain Monte Carlo settings
☒	☐	For hierarchical and complex designs, identification of the appropriate level for tests and full reporting of outcomes
☒	☐	Estimates of effect sizes (e.g. Cohen's d, Pearson's r), indicating how they were calculated

Our web collection on statistics for biologists contains articles on many of the points above.

Software and code

Policy information about availability of computer code

Data collection	Topspin 3.5 (Bruker Biospin); Image Lab 6.0 (BioRad); MO.Affinity Analysis 2 (Nanotemper)
Data analysis	Sparky 3.114 (UCSF), RNA_thread (ROSIE server); Pymol 2.1.1 (Schroedinger); Jalview 2.11

For manuscripts utilizing custom algorithms or software that are central to the research but not yet described in published literature, software must be made available to editors and reviewers. We strongly encourage code deposition in a community repository (e.g. GitHub). See the Nature Portfolio guidelines for submitting code & software for further information.

Data

Policy information about availability of data

All manuscripts must include a data availability statement. This statement should provide the following information, where applicable:

- Accession codes, unique identifiers, or web links for publicly available datasets
- A description of any restrictions on data availability
- For clinical datasets or third party data, please ensure that the statement adheres to our policy

All data needed to evaluate the conclusions in the paper are present in the paper and/or the Supplementary Materials. Model building is based on the publically available pdb files: 5lmn and 1y26. The derived model is publically available at SBGrid Database.

March 2021

Field-specific reporting

Please select the one below that is the best fit for your research. If you are not sure, read the appropriate sections before making your selection.

☒ Life sciences ☐ Behavioural & social sciences ☐ Ecological, evolutionary & environmental sciences

For a reference copy of the document with all sections, see nature.com/documents/nr-reporting-summary-flat.pdf

Life sciences study design

All studies must disclose on these points even when the disclosure is negative.

Sample size	The in vivo assays were performed as technical and biological triplicates. The in vitro assays were performed as technical triplicates on single samples. All NMR experiments were always at least performed as duplicates and always at least on biological duplicates.
Data exclusions	No data was excluded
Replication	Preparation of 30S and 30SDeltaS1 particles and rS1 protein was replicated at least three times and yielded the same results regarding purity, sample integrity and yield. In vitro transciption of riboswitch RNA was performed multiple times using different labelling schemes and always resulted in samples with identical secondary and tertiary structure, as verified by NMR spectroscopy, native and denaturating PAGE.
Randomization	Not relevant for this work as no clinical study was executed
Blinding	Not relevant for this work as no clinical study was executed

Reporting for specific materials, systems and methods

We require information from authors about some types of materials, experimental systems and methods used in many studies. Here, indicate whether each material, system or method listed is relevant to your study. If you are not sure if a list item applies to your research, read the appropriate section before selecting a response.

Materials & experimental systems

n/a	Involved in the study	
☐	☒	Antibodies
☒	☐	Eukaryotic cell lines
☒	☐	Palaeontology and archaeology
☒	☐	Animals and other organisms
☒	☐	Human research participants
☒	☐	Clinical data
☒	☐	Dual use research of concern

Methods

n/a	Involved in the study	
☒	☐	ChIP-seq
☒	☐	Flow cytometry
☒	☐	MRI-based neuroimaging

Antibodies

Antibodies used	6x His-AB (Thermo Fischer) 1:3000 in PBS buffer<br>https://www.thermofisher.com/order/genome-database/dataSheetPdf?<br>producttype=antibody&productsubtype=antibody_primary&productId=MA1-135-HRP&version=156
Validation	Antibody was tested on 30S ribosomes as negative control and on different proteins containing 6x or 10x His tag as positive control
